# Indoxyl Sulfate as a Mediator Involved in Dysregulation of Pulmonary Aquaporin-5 in Acute Lung Injury Caused by Acute Kidney Injury

**DOI:** 10.3390/ijms18010011

**Published:** 2016-12-23

**Authors:** Nozomi Yabuuchi, Masataka Sagata, Chika Saigo, Go Yoneda, Yuko Yamamoto, Yui Nomura, Kazuhiko Nishi, Rika Fujino, Hirofumi Jono, Hideyuki Saito

**Affiliations:** 1 Department of Clinical Pharmaceutical Sciences, Graduate School of Pharmaceutical Sciences, Kumamoto University, 1-1-1 Honjo, Chuo-ku, Kumamoto 860-8556, Japan; 159y1003@st.kumamoto-u.ac.jp (N.Y.); berry.of.kumamoto.antonio@gmail.com (M.S.); saigo@fc.kuh.kumamoto-u.ac.jp (C.S.); 157y3108@st.kumamoto-u.ac.jp (G.Y.); a-y.o.atemak_happiness7@docomo.ne.jp (Y.Y.); 18nomura@gmail.com (Y.N.); hjono@fc.kuh.kumamoto-u.ac.jp (H.J.); 2 Department of Pharmacy, Kumamoto University Hospital, 1-1-1 Honjo, Chuo-ku, Kumamoto 860-8556, Japan; fujinorica@gmail.com; 3 Department of Hemodialysis and Apheresis, Kumamoto University Hospital, 1-1-1 Honjo, Chuo-ku, Kumamoto 860-8556, Japan; kazuhiko-nishi@fc.kuh.kumamoto-u.ac.jp

**Keywords:** acute kidney injury, acute lung injury, indoxyl sulfate, aquaporin-5

## Abstract

High mortality of acute kidney injury (AKI) is associated with acute lung injury (ALI), which is a typical complication of AKI. Although it is suggested that dysregulation of lung salt and water channels following AKI plays a pivotal role in ALI, the mechanism of its dysregulation has not been elucidated. Here, we examined the involvement of a typical oxidative stress-inducing uremic toxin, indoxyl sulfate (IS), in the dysregulation of the pulmonary predominant water channel, aquaporin 5 (AQP-5), in bilateral nephrectomy (BNx)-induced AKI model rats. BNx evoked AKI with the increases in serum creatinine (SCr), blood urea nitrogen (BUN) and serum IS levels and exhibited thickening of interstitial tissue in the lung. Administration of AST-120, clinically-used oral spherical adsorptive carbon beads, resulted in a significant decrease in serum IS level and thickening of interstitial tissue, which was accompanied with the decreases in IS accumulation in various tissues, especially lung. Interestingly, a significant decrease in AQP-5 expression of lung was observed in BNx rats. Moreover, the BNx-induced decrease in pulmonary AQP-5 protein expression was markedly restored by oral administration of AST-120. These results suggest that BNx-induced AKI causes dysregulation of pulmonary AQP-5 expression, in which IS could play a toxico-physiological role as a mediator involved in renopulmonary crosstalk.

## 1. Introduction

Acute kidney injury (AKI), a syndrome recognized as the sudden deterioration of renal function from several hours to a few days, causes derangement of homeostatic maintenance of the body’s fluids and electrolytes [1]. AKI is characterized by increased levels of serum creatinine (SCr) and oliguria caused by functional or structural disturbances of the kidney, including abnormalities in blood, urine or tissues present for less than three months. Despite advances in understanding the pathophysiology, improvements in dialysis and supportive care, the mortality of AKI remains considerably high (ranging from 40% to 60%) [2]. The high mortality of AKI is associated with acute lung injury (ALI) or acute respiratory distress syndrome, which are typical complications of AKI [2]. Although it is well documented that lung injury is often associated with AKI and that lung dysfunction is highly correlated with death in patients with AKI [3], the mechanism underlying renopulmonary crosstalk has not been fully elucidated.

Several studies have suggested that dysregulation of lung salt and water channels following AKI plays a pivotal role in ALI [4,5]. Of various channels, it is known that aquaporin 5 (AQP-5) is a pulmonary predominant water channel and responsible for the majority of water transport across the apical membrane of type I alveolar epithelial cells [6,7]. A previous report has also shown that ischemic acute renal failure leads to downregulation of AQP-5 and the pulmonary epithelial sodium channel, Na^+^/K^+^-ATPase, and may modulate lung dysfunction and susceptibility to lung injury [8]. However, little is known about the mechanism of dysregulation of AQP-5 in the pathogenesis of ALI caused by AKI.

Uremic toxins, characterized as compounds retained as solutes in the serum that contribute to uremic syndrome, trigger a complex and variable symptomatology [9]. Indoxyl sulfate (IS), a putative low-molecular weight uremic toxin, is excreted in the urine under normal kidney function, but is retained in the blood circulation and various tissues during renal dysfunction in AKI and chronic kidney disease [10]. It is well documented that IS is exclusively generated in the liver through metabolic process by several hepatic metabolizing enzymes, such as sulfotransferase (SULT) 1A1 [11,12]. IS in the blood circulation is efficiently taken up by renal proximal tubular cells via basolateral membrane-localized organic anion transporters, OAT1/SLC22A6 and OAT3/SLC22A8, and excreted into the urine via unidentified apical membrane-localized transporters [13]. Our previous studies showed that the increase in the IS levels could be involved in the mechanism of the downregulation of renal organic ion transporters and central nervous system toxicities in cisplatin-induced AKI model rats [14,15]. It has also been shown that inhibition of IS production elicited a nephropreventive effect in the ischemic AKI model [16]. Moreover, various previous studies suggest that serum and tissue IS accumulation play crucial roles in the pathogenesis of AKI [17,18].

In this study, we developed the bilateral nephrectomy (BNx)-induced AKI rat model to examine the involvement of a typical oxidative stress-inducing uremic toxin, IS, in the dysregulation of the pulmonary predominant water channel, AQP-5, and elucidate the toxico-physiological role of IS as a mediator involved in renopulmonary crosstalk in the pathogenesis of ALI.

## 2. Results

### 2.1. SCr, BUN and Serum Accumulations of IS

**Figure 1:**
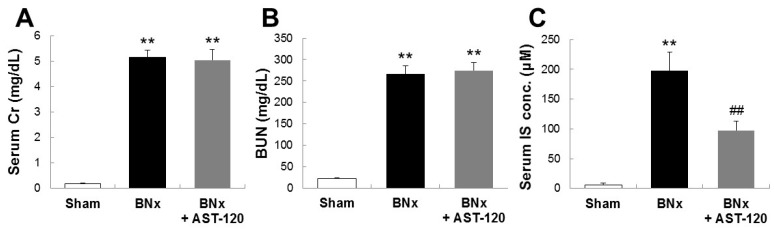
SCr, blood urea nitrogen (BUN) and serum accumulations of indoxyl sulfate (IS) in bilateral nephrectomy (BNx) rats. SCr (**A**), BUN (**B**) levels and serum IS concentration (**C**) were determined in sham-operated rats (sham), BNx rats with (BNx + AST-120) or without (BNx) oral administration of AST-120 (2.5 g/kg) at 48 h. Each column represents the mean ± SD for six to 11 rats in each group. ** *p* < 0.01 versus sham; ^##^ *p* < 0.01 versus BNx.

To determine the involvement of IS in the pathogenesis of ALI, we first sought to develop the bilateral nephrectomy (BNx)-induced AKI rat model. As shown in Figure 1, BNx evoked AKI with the increase in SCr, blood urea nitrogen (BUN) and serum accumulations of IS at 48 h. In addition, oral administration of AST-120, clinically-used oral spherical adsorptive carbon beads for reducing the accumulation of uremic toxins, resulted in a significant decrease in serum IS level (Figure 1C).

### 2.2. Organ Accumulation of IS and Histological Changes of Lung Tissue

**Figure 2:**
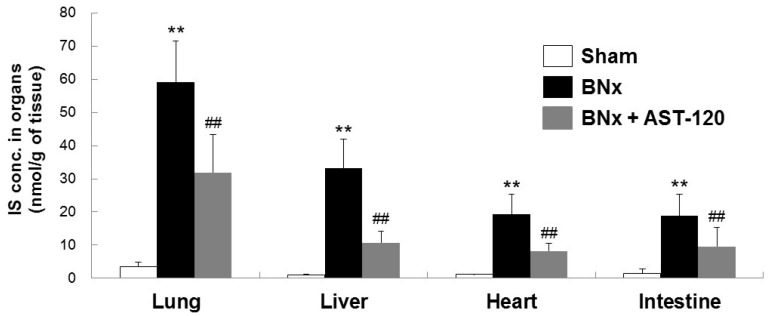
Organ accumulation of IS in BNx rats. Organ accumulation of IS were determined in sham-operated rats (sham), BNx rats with (BNx + AST-120) or without (BNx) oral administration of AST-120 (2.5 g/kg) at 48 h. Each column represents the mean ± SD for seven to 10 rats in each group. ** *p* < 0.01 versus sham; ^##^ *p* < 0.01 versus BNx.

**Figure 3:**
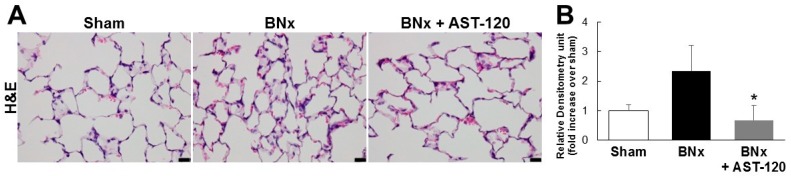
Histological changes of lung tissue in BNx rats. (**A**) H&E-stained sections of the lung tissue of sham-operated rats (sham), BNx rats with (BNx + AST-120) or without (BNx) oral administration of AST-120 (2.5 g/kg) at 48 h. Scale bars represent 20 μm; (**B**) Quantitative analysis of histological changes in lung tissue. Each column represents, the mean ± SD for three rats in each group. * *p* < 0.05 versus BNx.

Because BNx caused AKI with a significant increase in serum IS level, we next attempted to determine whether IS was accumulated in various kinds of tissue by using the BNx rat model. As shown in Figure 2, BNx significantly increased IS accumulation in various organs, especially lung tissue. Consistent with the result showing the significant decrease in serum IS level (Figure 1C), administration of AST-120 resulted in a significant decrease in IS accumulation in lung tissue (Figure 2). Moreover, in association with the marked decrease in IS accumulation in lung tissue, BNx-induced thickening of interstitial tissue in the lung was obviously suppressed by oral administration of AST-120 (Figure 3), suggesting that IS accumulation in lung tissue may play important roles in the pathogenesis of ALI.

### 2.3. AQP-5 and Na^+^/K^+^-ATPase Protein Expressions of the Lung

**Figure 4:**
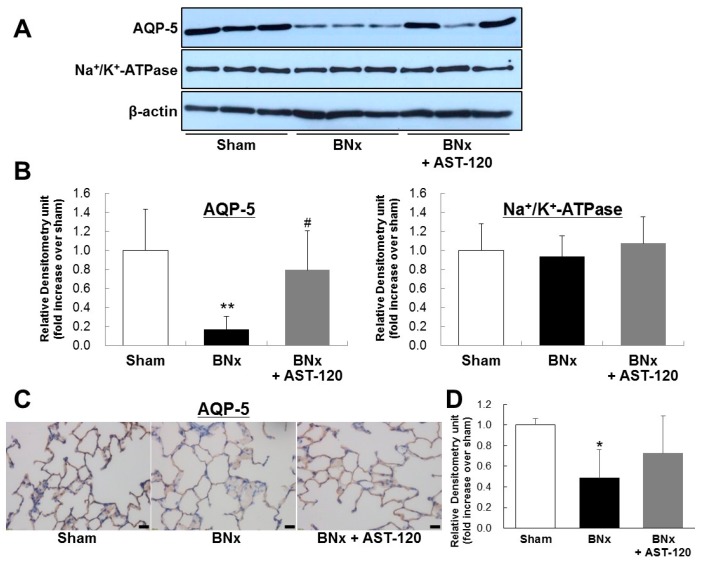
AQP-5 and Na^+^/K^+^-ATPase protein expressions of the lung in BNx rats. Protein expressions of AQP-5 and Na^+^/K^+^-ATPase of the lung in sham-operated rats (sham), BNx rats with (BNx + AST-120) or without (BNx) oral administration of AST-120 (2.5 g/kg) at 48 h, were determined by Western blot analysis (**A**,**B**) and immunohistochemical analysis (**C**) with antibody against rAQP-5. Each column represents the mean ± SD from three rats. ** *p* < 0.01 versus sham; ^#^ *p* < 0.05 versus BNx. Scale bars = 20 μm; (**D**) Quantitative analysis of AQP-5 protein expression in lung tissue. Each column represents the mean ± SD for three rats in each group. * *p* < 0.05 versus sham.

It has been reported that dysregulation of lung salt and water channels following AKI plays a pivotal role in ALI [4,5]. To determine the involvement of IS in dysregulation of pulmonary predominant water channels in ALI, we next examined AQP-5 and Na^+^/K^+^-ATPase protein expressions of lung in BNx rats. Western blot analysis showed the significant decrease in AQP-5 expression of lung in BNx rats (Figure 4A,B). Interestingly, BNx-induced significant decrease in AQP-5 expression was obviously restored by oral administration of AST-120 (Figure 4A,B). By contrast, no significant change of Na^+^/K^+^-ATPase protein expression was observed. Moreover, immunohistochemical analysis confirmed that BNx-induced decrease in AQP-5 protein expression in lung tissue was also restored by oral administration of AST-120 (Figure 4C,D), suggesting that IS accumulation in lung tissue may play crucial roles in the pathogenesis of ALI through dysregulation of pulmonary AQP-5 expression.

## 3. Discussion

Despite advances in understanding the pathophysiology, improvements in dialysis and supportive care, the mortality of AKI remains considerably high [2]. Although the high mortality of AKI is associated with ALI, which is a typical complication of AKI, the molecular pathogenesis of ALI has yet to be determined. In the present study, we showed that BNx-induced AKI caused IS accumulation in the lung tissue, which in turn may lead to ALI progression via dysregulation of pulmonary AQP-5 expression.

One of the interesting finding in this study is that IS may play a toxico-physiological role as a mediator involved in renopulmonary crosstalk. It has been reported that IS concentrations in lung were markedly elevated in both 5/6 nephrectomized rats and cisplatin-induced AKI rats [19,20]. Consistent with those previous reports, our results also showed that, in association with the marked increase in serum IS concentration, BNx significantly increased IS accumulation in various organs, especially lung tissue (Figure 2). It is to be noted that the significant increase in serum accumulations of IS was observed at 4 h (Figure S1A). Moreover, IS accumulation in lung already showed a tendency to increase at 4 h (Figure S1B). It is known that AST-120 can reduce the accumulation of uremic toxins, such as IS, by restriction of protein intake in the intestine [21,22]. Because oral administration of AST-120 indeed restored BNx-induced thickening of interstitial tissue following IS accumulation in the lung (Figure 3), ALI progression may be associated with the increase in serum IS concentration caused by renal injury through renopulmonary crosstalk. It should be noted that SCr and BUN were not restored by AST-120 treatment (Figure 1A,B), since there was likely no renoprotective effect caused by decreasing serum IS concentration due to bilateral nephrectomy.

A previous report has shown that ischemic acute renal failure leads to downregulation of the pulmonary epithelial sodium channel, Na^+^/K^+^-ATPase and AQP-5 and may modulate lung dysfunction and susceptibility to lung injury [8]. Those findings suggest that dysregulation of lung salt and water channels following AKI may play a pivotal role in ALI [3,4,5]. Our results showed that IS accumulation triggered the dysregulation of AQP-5 protein expression in lung tissue (Figure 4). Moreover, both Western blot and immunohistochemical analysis showed that the BNx-induced significant decrease in pulmonary AQP-5 expression was obviously restored by oral administration of AST-120 (Figure 4), suggesting that IS accumulation in lung tissue may play pivotal roles in the pathogenesis of ALI through dysregulation of pulmonary AQP-5 expressed in alveolar epithelial cells. Meanwhile, no significant change of Na^+^/K^+^-ATPase protein expression was observed in BNx rats. Since it has been reported that Na^+^/K^+^-ATPase protein expression was significantly changed by ischemic acute renal failure [8], regulation of Na^+^/K^+^-ATPase protein expression is likely mediated by ischemia-related factors, such as inflammatory mediators, rather than uremic toxins. Several studies have shown that IS accumulation is associated with not only dysregulation of lung water channels, but also various risk factors, such as reactive oxygen species, transforming growth factor-β1, tissue inhibitor of metalloproteinase-1, intracellular adhesion molecule-1 and plasminogen activator inhibitor-1 [23,24,25,26,27]. In addition, it has been reported that IS upregulates monocyte chemotactic protein-1 expression through the production of reactive oxygen species and activation of the MAPK and JNK pathway [28]. Because it is also documented that p38 MAPK and JNK activation downregulate AQP5 expression in alveolar epithelial cells [29], IS accumulation may cause dysregulation of pulmonary AQP-5 expression by activating the p38 MAPK and JNK pathway, which in turn leads to thickening of interstitial tissue in the lung. Moreover, alveolar epithelium expressed not only AQP-5, but also AQP 3 and AQP4 [30]. Therefore, future studies will focus on determining the molecular mechanism of IS-induced dysregulation of AQP-5 and also further exploring the other factors involved in IS-induced ALI.

It is documented that IL-6 contributes to AKI-mediated lung injury, potentially via effects on lung production of chemokines [31]. In addition to the fact that IL-6 was significantly elevated in patients with AKI, circulating IL-6 levels could be used as a prognostic marker in patients with ALI [31]. To further determine the involvement of IL-6 in IS accumulation and dysregulation of pulmonary AQP-5, we measured serum IL-6 concentration in BNx rats at 4 h, which showed the highest serum IL-6 levels in a previous study [32]. As shown in Figure S2A, BNx significantly increased serum accumulations of IS, and AST-120 treatment decreased its serum IS levels even at 4 h. However, BNx-induced serum IL-6 elevation was not suppressed by AST-120 treatment (Figure 2B), suggesting that IL-6 may be involved in the pathogenesis of ALI independently of IS. Based on the previous studies, IL-6 may cause ALI progression through histological damage or neutrophil infiltration [27], rather than dysregulation of pulmonary AQP-5.

In conclusion, our results suggest that IS accumulation in lung tissue may play crucial roles in the pathogenesis of ALI in BNx rats. BNx-induced AKI caused dysregulation of pulmonary AQP-5 expression, in which IS could play a toxico-physiological role as a mediator involved in renopulmonary crosstalk. This finding may bring new insights into understanding of ALI pathogenesis and may provide useful basic information for establishing a new therapeutic strategy to prevent the high mortality of AKI associated with ALI.

## 4. Materials and Methods

### 4.1. Chemicals

IS was obtained from Sigma-Aldrich Co. (St. Louis, MO, USA). AST-120 was kindly provided by Daiichi Sankyo Co., Ltd. (Tokyo, Japan). Carboxymethyl cellulose (CMC) and methanol were obtained from Wako Pure Chemical Industries, Ltd. (Osaka, Japan). All chemicals used in this study were of analytical grade and commercially available.

### 4.2. Animal Experiments

All procedures for animal experiments were approved by the Kumamoto University ethical committee concerning animal experiments (Identification code: A 27-045, Approval date: 01/04/2015) and animals were treated in accordance with the Guidelines of the United States National Institutes of Health regarding the care and use of animals for experimental procedures and the Guidelines of Kumamoto University for the care and use of laboratory animals. Male Sprague–Dawley (SD) rats at 6 weeks of age were housed in a standard animal maintenance facility at a constant temperature (22 ± 2 °C) and humidity (50%–70%) and a 12/12-h light/dark cycle for about a week before the day of the experiment, with food and water available ad libitum. Rats were anesthetized using sodium pentobarbital (50 mg/kg intraperitoneally) and placed on a heating plate (39 °C) to maintain a constant temperature. All surgery was conducted under anesthesia with pentobarbital, and all efforts were made to minimize animal suffering. The kidneys of male SD rats at 6 weeks of age were exposed via midline abdominal incisions. In the bilateral nephrectomy model, both renal pedicles were tied off with a suture and then cut distal to the suture. The ureters were pinched off with forceps, and the kidneys were removed as previously reported [33]. Sham animals (control) underwent anesthesia, laparotomy and renal pedicle dissection only. Rats were divided into three different groups as follows: sham-operated rats (control rats), CMC-administered rats with BNx and AST-120-administered rats with BNx. AST-120 (2.5 g/kg) was orally administered to rats 24 and 1 h before and 24 h after BNx. Blood was collected 4 or 48 h after BNx from the abdominal aorta and centrifuged at 3000× *g* for 10 min to obtain the serum sample. Methanol (100 μL) was added to serum (50 μL), and the mixture was centrifuged at 13,000 rpm for 10 min at 4 °C. The obtained supernatant (50 μL) was diluted with HPLC mobile phase solution (300 μL) and centrifuged at 13,000 rpm for 5 min at 4 °C. The supernatant was used for HPLC determination of IS concentration. Lung, liver, heart and intestine was harvested 48 h after BNx and homogenized in phosphate-buffered saline (pH 7.4) using a Polytron PT3000 (Kinematica AG, Lucerne, Switzerland). After centrifugation at 3000 rpm for 10 min at 4 °C, the obtained supernatant was used for the HPLC assay of IS concentration. Lung samples were fixed in 10% buffered formaldehyde and embedded in paraffin for H&E staining and immunohistochemistry. Levels of SCr (enzymatic method) and BUN (uricase ultraviolet (UV) method) were then measured.

### 4.3. High-Performance Liquid Chromatography Determination of IS Concentration

HPLC was performed according to a previous report with some modifications [14,34]. The HPLC system consisted of a Shimadzu LC-10ADVP pump and a Shimadzu RF-10AXL fluorescence spectrophotometer. A column of LiChrospher^®^ 100 RP-18 (Merck KGaA, Darmstadt, Germany) was used as the stationary phase, and the mobile phase consisted of acetate buffer (0.2 M, pH 4.5). The flow rate was 1.0 mL/min at a column temperature of 40 °C. The presence of IS in the eluate was monitored by means of a fluorescence detector (excitation 280 nm, emission 375 nm).

### 4.4. Western Blot Analysis

Western blot was performed according to a previous report with some modifications [16,19,35]. Kidneys were homogenized in an ice-cold homogenization buffer consisting of 230 mM sucrose, 5 mM Tris (hydroxymethyl) aminomethane hydrochloride (Tris–HCl) (pH 7.5), 2 mM ethylenediaminetetraacetic acid, 0.1 mM phenylmethanesulfonyl fluoride, 1 μg/mL leupeptin and 1 μg/mL pepstatin A. After measuring protein content using a bicinchoninic acid (BCA) protein assay reagent (Thermo Fisher Scientific, Waltham, MA, USA), each sample was mixed in loading buffer (2 *w*/*v* % sodium dodecyl sulfate (SDS), 125 mM Tris-HCl pH 7.2, 20 *v*/*v* % glycerol and 5 *v*/*v* % 2-mercaptoethanol) and heated at 95 °C for 2 min. The samples were subjected to sodium dodecyl sulfate-polyacrylamide gel electrophoresis using a 7.5% gel and transferred onto a polyvinylidene difluoride membrane (Immobilon-P; EMD Millipore, Billerica, MA, USA) by semi-dry electroblotting. The membrane was blocked for 1 h at room temperature with 2 *v*/*v* % ECL Advance Blocking Agent (GE Healthcare UK Ltd., Little Chalfont, UK) in 50 mM Tris-buffered saline (pH 7.6) containing 0.3 *v*/*v* % Tween 20, and then incubated for 1 h at room temperature with a primary antibody specific for rAQP-5 (Alpha Diagnostic, San Antonio, TX, USA) or Na^+^/K^+^-ATPase (upstate biotechnology, Inc., Lake Placid, NY, USA). The blots were then washed with Tris-buffered saline containing Tween 20 before incubation with the secondary antibody (horseradish peroxidase-labeled anti-rabbit immunoglobulin F(ab)2 or horseradish peroxidase-linked anti-mouse immunoglobulin F(ab)2) (GE Healthcare Ltd., Chicago, IL, USA) for 1 h at room temperature. Immunoblots were visualized with an ECL system (ECL Advance Western Blotting Detection Kit; GE Healthcare Ltd., Chicago, IL, USA).

### 4.5. Histochemical Staining

Histochemical staining was performed according to a previously-described report with some modifications [16,36,37]. Paraffin-embedded specimens were cut into 6-µm sections and mounted on glass slides. After deparaffinization of the sections, rehydration, pretreatment with a microwave for 2 × 10 min in citrate buffer pH 6.0, washing in phosphate-buffered (PBS), pH 7.4, were followed by the blocking of endogenous peroxidase with 0.3% H_2_O_2_ for 15 min and a blocking in Blocking One Histo (Nacalai tesque, Kyoto, Japan) for 15 min at room temperature. The primary antibody specific for rAQP-5 (Alpha Diagnostic, San Antonio, TX, USA) was incubated overnight at 4 °C, followed by washings in PBS. The secondary antibody (horseradish peroxidase-labeled anti-rabbit immunoglobulin F(ab)2) was incubated for 1 h at room temperature, followed by washings in PBS. DAB solution (Dako, Tokyo, Japan) was then added for coloration for 15 min at room temperature, followed by counterstaining with hematoxylin. The sections were deparaffinized and stained with hematoxylin-eosin (H&E). Pathological changes of lung tissue in BNx rats were assessed by hemorrhage, a hallmark of ALI associated with increased lung vascular permeability [38]. Quantitative analysis of immunohistochemical images and pathological changes of lung tissue were performed using WinRoof V7.4 (MITANI Corporation, Tokyo, Japan), which performed automated particle analysis in a measured area as described previously [39].

### 4.6. Statistical Analysis

Data were analyzed statistically by analysis of variance, followed by Scheffé’s multiple comparison test. A *p*-value of <0.05 was considered statistically significant. All data are represented as the mean ± standard deviation (SD).

## Supplementary Materials

Supplementary materials can be found at www.mdpi.com/1422-0067/18/1/11/s1.
