# Supplementary material for: Indoxyl Sulfate as a Mediator Involved in Dysregulation of Pulmonary Aquaporin-5 in Acute Lung Injury Caused by Acute Kidney Injury
Source: Int J Mol Sci. 2016 Dec 23;18(1):11. doi: 10.3390/ijms18010011 (PMC5297646; doi:10.3390/ijms18010011)
Supplement: Supplementary file 1 [file ijms-18-00011-s001.pdf]

Supplementary Materials: Indoxyl Sulfate as a Mediator Involved in Dysregulation of Pulmonary Aquaporin-5 in Acute Lung Injury Caused by Acute Kidney Injury

Nozomi Yabuuchi, Masataka Sagata, Chika Saigo, Go Yoneda, Yuko Yamamoto, Yui Nomura, Kazuhiko Nishi, Rika Fujino, Hirofumi Jono and Hideyuki Saito

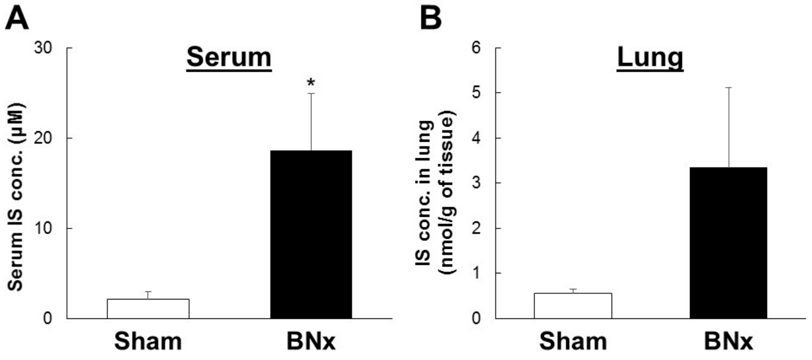

Figure S1. Accumulations of IS in serum and lung tissue at 4 h. Serum IS concentration (**A**); and IS accumulation in lung (**B**) were determined in sham-operated rats (sham) and BNx rats at 4 h. Each column represents the mean ± S.D. * $p < 0.05$ versus sham.

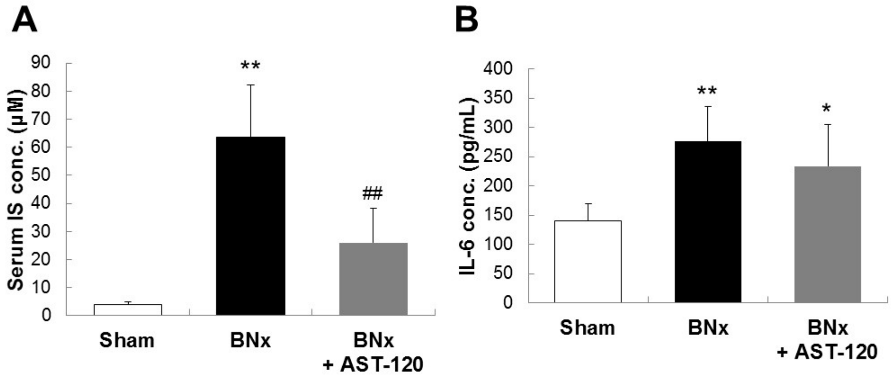

Figure S2. Serum accumulations of IS and IL-6 concentration in BNx rats at 4 h. Serum IS concentration (**A**); and IL-6 concentration (**B**) were determined in sham-operated rats (sham), BNx rats with (BNx + AST-120) or without (BNx) oral administration of AST-120 (2.5 g/kg) at 4 hr. Each column represents the mean ± S.D. for 6 to 11 rats in each group. ** $p < 0.01$ versus sham; * $p < 0.05$ versus sham; ## $p < 0.01$ versus BNx.
